# A Robust Prognostic Gene Signature Based on eRNAs-Driven Genes in Prostate Cancer

**DOI:** 10.3389/fgene.2021.676845

**Published:** 2021-06-29

**Authors:** Shuaishuai Fan, Zheng Wang, Li Zhao, ChenHui Zhao, DaJiang Yuan, Jingqi Wang

**Affiliations:** ^1^First Clinical Medical College, Shanxi Medical University, First Hospital of Shanxi Medical University, Taiyuan, China; ^2^People’s Hospital of Zezhou County, Jincheng, China; ^3^Department of Anesthesia, Shanxi Medical University, Taiyuan, China; ^4^The First People’s Hospital of Jinzhong, Jinzhong, China

**Keywords:** prostate cancer, enhancer RNA, prognostic gene signature, risk score model, drug sensitivity

## Abstract

Prostate cancer (PCa) is the second most common malignancy in men, but its exact pathogenetic mechanisms remain unclear. This study explores the effect of enhancer RNAs (eRNAs) in PCa. Firstly, we screened eRNAs and eRNA -driven genes from The Cancer Genome Atlas (TCGA) database, which are related to the disease-free survival (DFS) of PCa patients;. screening methods included bootstrapping, Kaplan–Meier (KM) survival analysis, and Pearson correlation analysis. Then, a risk score model was established using multivariate Cox analysis, and the results were validated in three independent cohorts. Finally, we explored the function of eRNA-driven genes through enrichment analysis and analyzed drug sensitivity on datasets from the Genomics of Drug Sensitivity in Cancer database. We constructed and validated a robust prognostic gene signature involving three eRNA-driven genes namely *MAPK15, ZNF467*, and *MC1R*. Moreover, we evaluated the function of eRNA-driven genes associated with tumor microenvironment (TME) and tumor mutational burden (TMB), and identified remarkable differences in drug sensitivity between high- and low-risk groups. This study identified a prognostic gene signature, which provides new insights into the role of eRNAs and eRNA-driven genes while assisting clinicians to determine the prognosis and appropriate treatment options for patients with PCa.

## Introduction

Prostate cancer (PCa) is the second most common malignancy in men (No Authors Listed, 2020). At present, the main treatment for primary PCa is radical prostatectomy (Albertsen, 2020), and although most patients with PCa benefit from this procedure, the recurrence rate following this treatment remains high (27–53%) (Van den Broeck et al., 2019). Almost all recurrent PCa will develop into advanced PCa and further progress into castration-resistant prostate cancer (CRPC). For advanced PCa and CRPC, targeted therapy and chemotherapy are the main treatment options of disease management (Connor et al., 2021). Therefore, exploring the mechanisms involved in PCa and identifying effective PCa biomarkers, are important measures to improve the prognosis of patients with PCa.

Enhancer RNAs (eRNAs)—important components of long non-coding RNAs (lncRNAs)—are transcribed from enhancer regions and important in gene transcriptional regulation (Sedano et al., 2020). Numerous eRNAs have been found in human cells, which occupy a high position in the transcriptional circuit by mediating the activation of target genes (Ding et al., 2018). eRNAs, induced by tumor suppressors, could suppress tumors (Wang et al., 2020). On the contrary, oncogene-induced eRNAs can directly promote tumorigenesis (Liang et al., 2016). Zhang et al. (2019) also showed that eRNAs play an important role in the development and treatment of tumors. These findings illustrate the importance of eRNAs in the process of human oncogenesis.

**FIGURE 1:**
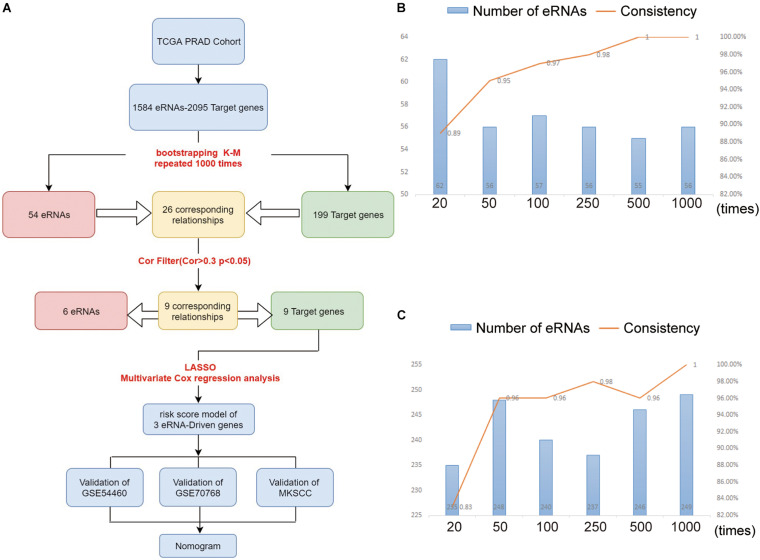
**(A)** The workflow of the study. The eRNAs **(B)** and target genes **(C)** results of nodes 10, 40, 90, 240, 490, 990 compared with nodes 20, 50, 100, 250, 500, and 1,000. As the number of repetitions increased, we compared the results at the node with the previous 10 times, and found that the consistency of the results increased continuously. At the level of 1,000 repetitions, the consistency reached about 100%.

With the advent of high-throughput sequencing, it is possible to explore the function of eRNAs across the whole genome. Previous studies have conducted preliminary investigations of the functions of eRNAs. For example, Xiaolian et al. found that AP001056.1—a key immune-related eRNA in head and neck squamous cell carcinoma (SCCHN)—has a positive effect on clinical outcomes in patients with SCCHN (Liang et al., 2016). However, the role of eRNAs in PCa is not clear. Therefore, we used high-throughput sequencing technology and bioinformatics to screen eRNAs and target genes that may influence the prognosis of patients with PCa and constructed a robust prognostic model. Moreover, we explored the mechanism of eRNAs’ influence on PCa development, as well as the predictive power of the model for PCa drug sensitivity and potential PCa drug identification. We also investigated the functions of prognostic genes associated with PCa using a multi-omics, multi-database approach involving various methods. The workflow of our research is shown in Figure 1A. In total, 835 prostate samples were included, and the pathogenesis and treatment of PCa was explored.

## Materials and Methods

### Data Collection and Pre-processing

The transcriptome data of PCa and paracancerous tissues, as well as clinical data of patients with PCa as per The Cancer Genome Atlas (TCGA) database, were obtained from the National Institutes of Health (NIH) Genomic Data Commons (GDC Data Portal^1^). Using the Gene Expression Omnibus (GEO^2^) and the cBioPortal (cBio^3^), we searched for PCa datasets that included: (1) PCa and RNA-seq or micro-array dataset types; (2) more than 100 PCa samples in survival data; and (3) information on the expression of the genes in the gene signature. Per these parameters we obtained two datasets, namely GSE54460 and GSE70768 from the GEO and one dataset, MSKCC (Cerami et al., 2012), from cBio. We also obtained data on the lncRNA-target gene relationships, as predicted by PreSTIGE (Predicting Specific Tissue Interactions of Genes and Enhancers), and the lncRNA expressions from active tissue-specific enhancers. Using the human gene annotation file and Perl, we converted the Ensembl ID to its corresponding transcript ID and gene name, respectively. Using the Tumor Immune Estimation Resource 2.0 (TIMER2.0), we downloaded the immune cell infiltration data of prostate adenocarcinoma (PRAD) tissues from the Cistrome Project^4^. We obtained the gene sets associated with epithelial-mesenchymal transformation (EMT), transforming growth factor β (TGF-β), and extracellular matrix (ECM) from the Molecular Signatures Database (MSigDB^5^) (Zhang et al., 2020). The copy number alterations (CNAs) analysis was performed by cBio.

### Screening of eRNAs and Target Genes Associated With the Prognosis of Patients With PCa

To preliminarily screen eRNAs related to the prognosis of patients with PCa, we analyzed the eRNAs and their expression levels along with their corresponding clinical disease-free survival (DFS) rates. Through application of bootstrapping methods and the Kaplan–Meier (KM) survival analysis, candidate eRNAs were narrowed down as follows: 70% of the samples were randomly selected from the TCGA data cohort for gene survival analysis. The process was repeated 1,000 times. Genes that appeared more than 700 times among the samples (robustness test, *P* < 0.05) were regarded as robust prognostic genes (Zhou et al., 2019). The target genes related to prognosis were screened using the same method. Based on the correlation analysis between the eRNAs and target genes, we suggested that the target genes (cor > 0.3; *P* < 0.05; CNAs of target genes were no significant correlation with its corresponding eRNAs) are defined as eRNA-driven genes.

### Building the Predictive Signature for Patients With PCa

For the training cohort from TCGA dataset, we adopted a least absolute shrinkage and selection operator (LASSO) regression analysis using the R package glmnet 4.0-2 (Friedman et al., 2010; Simon et al., 2011), to narrow down the range of eRNA-driven genes related to the prognosis of patients with PCa. Using the R package survival 3.2-7, we developed a risk model through performing multivariate Cox regression analysis. Applying the formula:

risk score = $\sum_{k=1}^{n}\left(\mathrm{risk}\,\mathrm{genes}\,{\mathrm{expression}}_{k}\times {\mathrm{coefficient}}_{k}\right)$, we were able to obtain the prognostic risk scores.

### Assessment and Validation of the Risk Score Model

Due to the differences between the sequencing data and the chip data, the risk scores of the training set (TCGA) and the Validation sets (GSE55460, GSE70768, and MSKCC) were calculated, respectively, according to the above risk scoring calculation formula. Then, using the median value of their risk scores, we divided the training set and the Validation sets into high- and low-risk groups, respectively. We further used the survival data of the two groups of patients with PCa, to generate KM survival curves through a log-rank test (the model has predictive value when *P* < 0.05). Using the R package survivalROC 1.0.3 (Kamarudin et al., 2017), we conducted a time-dependent receiver operating characteristic (ROC) analysis to determine the accuracy of the prediction model. An area under the curve (AUC) of > 0.60 was considered to reflect minimum predictive value, while AUC > 0.75 showed good predictive value.

### Enrichment Analysis of Prognostic Genes in PCa

We identified the differentially expressed genes (DEGs)in high- and low-risk groups from TCGA cohort using the R package limma 3.42.2. An absolute log2 fold change (| FC|) > 1 and an adjusted false discovery rate (FDR) < 0.05 were set as cutoff criteria. Using the R package limma 3.42.2, 609 DEGs were subjected to Gene Ontology (GO) enrichment analysis. Furthermore, Gene Set Enrichment Analysis (GSEA) was conducted using GSEA 3.0, per the methods specified in the user guide (gsea-3.0.jar^6^).

### Screening of Prognostic Clinical Factors in PCa

Using the R package survival 3.2-7, we performed univariate Cox regression analysis to appraise the predictive value of the risk score and other clinical factors for DFS in patients with PCa. Then, we conducted multivariate Cox regression analysis to remove confounders. The bilateral significance level was 0.05, and the hazard ratio (HR) and 95% confidence interval (CI) were calculated.

### Establishment and Evaluation of the Nomogram

Combining the prognostic factors analyzed using the above methods, we used the R package rms 6.1-0 to construct a nomogram. Using the R package pec v2019.11.3 (Mogensen et al., 2012), we performed Harrell’s concordance index (C-index) analysis to assess the performance of the nomogram. A robust C-index was obtained using 1,000 bootstrap resamples. The closer the score was to 0.5, the less discriminative it was deemed to be, and vice versa for scores closer to 1. The efficiency of the nomogram was evaluated using the time-dependent ROC curve.

### Drug Sensitivity Analysis

We used R package pRRophetic to compare half maximal inhibitory concentrations (IC50) of two drugs (docetaxel, bicalutamide) used in treatment of PCa, as per the Genomics of Drug Sensitivity in Cancer database. Results were recorded for, and compared between, the high- and low-risk groups. In addition, we used the Connectivity Map (CMap)^7^ to screen small molecule drugs that are beneficial to the prognosis of PCa. We obtained the *P* value of the association between each small molecule compound and the differential genes, by using the CMap online tool (Ma et al., 2021); *P* < 0.05 was statistically significant.

### Statistical Analysis

All statistical analyses were performed using R software (version 3.6.3). The Mann–Whitney *U* test was used to compare two groups with non-normally distributed variables. Furthermore, Kruskal–Wallis analysis of variance was applied as non-parametric method to compare the three groups. All statistical tests were two-sided; *P* < 0.05 was statistically significant.

## Results

### Identification of Prognosis-Associated eRNAs and Target Genes in PCa

Table 1 summarizes the important clinical factors from TCGA data involved in the study. Based on previous findings (Vučićević et al., 2015), we constructed a table of the relevant eRNAs and their predicted target genes. Through bootstrapping, we performed KM survival analysis on 1,584 eRNAs and 2,095 eRNA-driven genes. Finally, we identified 54 eRNAs and 199 target genes as prognosis-associated genes (Supplementary Table 1). With this screening method, randomness was significantly removed and the stability of results improved.

**TABLE 1:** Clinical information from the 545 PCa patients of TCGA.

Clinical parameters	Variable	Number (total)	Percentage	Hazard ratio
Age (years)	≤60	242	44.40%	1.338 (0.874–2.047)
	>60	303	55.60%	
Survival status	Dead	10	1.83%	Not applicable
	Alive	482	88.44%	
	Unknown	53	9.72%	
Gleason score	6	48	8.81%	3.907 (2.471–6.178)
	7	285	52.29%	
	8	66	12.11%	
	9 and 10	146	26.78%	
T grade	T2	188	34.50%	4.018 (2.224–7.256)
	T3	295	54.13%	
	T4	10	1.83%	
	Unknown	52	9.54%	
N grade	N0	348	63.85%	1.820 (1.112–2.980)
	N1	79	14.50%	
	Unknown	118	21.65%	

In order to verify that the Bootstrap method is more rational, we set six nodes (20, 50, 100, 250, 500, and 1,000) and compared the results at each node as shown in Figures 1B,C. Node 20 indicates the 20th time of Bootstrap, while node 50 indicates the 50th time, and so on. Taking node 20 as an example, we compared the result at the 20th-time Bootstrap with that of 10th time. For each the 10th and 20th times, we identified 66 genes for further screening and found that intersection for 62 genes when comparing the two nodes (we defined the intersection gene number as N). The unique gene number of the 10th time is defined as X, while that of the 20th time as Y. From these results, we defined the value of N/(X + Y + N) as the consistency of the two results. As shown in Figures 1B,C, as the number of repeats increases, the consistency of data at the nodes increases to 100%, while the number of intersected genes gradually tends to be stable.

### Acquisition of Candidate Genes and Labeling of Gene Function

Through comparative examinations, we screened 26 regulatory relationships and performed correlation analysis of these relationships (cor > 0.3, *P* < 0.05). We identified nine relationships consisting of six eRNAs and nine target genes (Table 2 and Supplementary Figure 1), from which we obtained nine candidate genes of which the functions were annotated using the Uniport database^8^ (Supplementary Table 2).

**TABLE 2:** List of eRNAs and eRNAs-driven genes associated with overall survival derived from enhancers.

Ensembl ID	Symbol	TANRIC Overall Survival Analysis, Log-Rank *p*-Value	Target gene	Correlation between lncRNA and the Neighboring Target	
	
				*p*-value	Correlation coefficient
ENSG00000223959	AFG3L1P	0.004207	FANCA	*P* < 0.001	0.418196
ENSG00000223959	AFG3L1P	0.004207	MC1R	*P* < 0.001	0.70217
ENSG00000223959	AFG3L1P	0.004207	SPIRE2	*P* < 0.001	0.556318
ENSG00000161912	ADCY10P1	4.72E-05	UNC5CL	*P* < 0.001	0.64206
ENSG00000206417	H1FX-AS1	7.75E-06	RPL32P3	*P* < 0.001	0.372866
ENSG00000242258	LINC00996	0.002828	GIMAP4	*P* < 0.001	0.678696
ENSG00000254812	AC067930.3	0.006284	MAPK15	*P* < 0.001	0.435871
ENSG00000197558	SSPOP	0.000238	ZNF467	*P* < 0.001	0.603343
ENSG00000227297	SSPOP	0.000863	ZNF862	*P* < 0.001	0.68106

### Building the Predictive Signature for PCa

**FIGURE 2:**
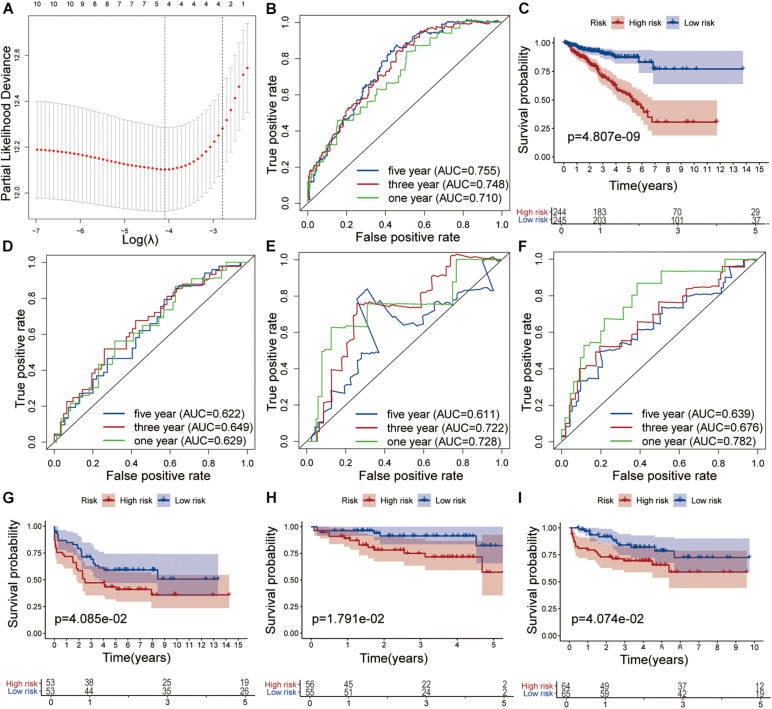
Development and validation of the risk model. **(A)** Tuning parameter (λ) selection in the LASSO model used 10-fold cross-validation via the maximum criteria. Time-dependent ROC curve analysis of the prognostic model in the TCGA cohort **(B)**, in the GSE54460 cohort **(D)**, in the GSE70768 cohort **(E)** and in the MKSCC cohort **(F)**. Kaplan-Meier curve analysis of high-risk the low-risk groups in the TCGA cohort **(C)**, in the GSE54460 cohort **(G)**, in the GSE70768 cohort **(H)** and in the MKSCC cohort **(I)**.

The nine target genes were filtered using LASSO analysis. The filter condition was set to repeat 1,000 times (Figure 2A). Therefore, the number of candidate genes was narrowed down from nine to three. We used the three screened genes in the multivariate Cox regression analysis (without bidirectional stepwise regression) to establish a prognostic model for PCa (Table 3). For each of the three genes, the product of the correlation coefficient and gene expression was used to determine the relevant risk score. Thus, we established a risk score model that calculated the risk score as follows: risk score = (0.186338 × *MAPK15* gene expression) + (0.523366 × *ZNF467* gene expression) + (0.624495 × *MC1R* gene expression). Correlation analysis between CNVs of model genes and its corresponding eRNAs showed no significant correlation among them (cor < 0.3) (Supplementary Figures 3A–C).

**TABLE 3:** Risk genes in the prognostic risk model.

Gene	Coef	HR	HR.95L	HR.95H	*p* value
MAPK15	0.186338	1.204829	0.977376	1.485215	0.080882
ZNF467	0.523366	1.687699	1.345957	2.116209	5.80E-06
MC1R	0.624495	1.867304	1.332502	2.616749	0.000286

### Evaluation of the Prognostic Risk Score Model for PCa

The AUC values for 1-, 3-, and 5-year survival were 0.710, 0.748, and 0.755, respectively (Figure 2B). Using the median risk score (0.951), we distributed the 489 samples with complete clinical data in TCGA cohort into a high-risk group consisting of 244 samples and a low-risk group of 245. The DFS of the low-risk group was significantly higher than that of the high-risk group (log-rank test, *P* < 0.05, Figure 2C).

We also analyzed the performance of individual model genes in the training set and showed that the AUC values for 1-, 3-, and 5-year survival of MAPK15 were 0.671, 0.718, and 0.731, respectively (Supplementary Figure 4A), while those of ZNF467 were 0.631, 0.715, and 0.719, respectively (Supplementary Figure 4B), and of MC1R were 0.669, 0.634, and 0.626, respectively (Supplementary Figure 4C). No significant correlation between the three genes was observed (Supplementary Figure 4D). The results suggest that the predictive value of the model is better than that of a single gene.

### Validation of the Prognostic Risk Score Model for PCa

The prognostic risk model was further validated in the GEO datasets GSE54460, GSE70768, and MSKCC. The clinical information of the validation cohort is summarized in Table 4. As shown in Figure 2D, the prognostic risk score model performed well in GSE54460, and the AUC values for 1-, 3-, and 5-year survival were 0.629, 0.649, and 0.622, respectively. Using the median risk score for segmentation (0.502), we distributed the 106 samples into high- and low-risk groups, containing 53 samples each. This result was similar to that obtained in the KM analysis of the training cohort (Figure 2G). In GSE70768, the respective AUC values for 1-, 3-, and 5-year survival were 0.728, 0.722, and 0.611, respectively (Figure 2E). Similarly, using the median risk score for segmentation (9.773), we divided the 111 samples into a low-risk group of 55 and a high-risk group of 56. In the KM analysis of GSE70768, the results of the low-risk group were significantly different from those of the high-risk group (log-rank test, *P* < 0.05, Figure 2H). In MSKCC, the AUC values for 1-, 3-, and 5-year survival were 0.782, 0.676, and 0.639, respectively. Using the median risk score (4.210) as discriminator, we divided the 129 samples into two groups of which 64 were deemed as low-risk, and 65 as high-risk. In the KM analysis of MSKCC, the results of the low-risk group were also significantly different from those of the high-risk group (log-rank test, *P* < 0.05, Figures 2F,I).

**TABLE 4:** Clinical recurrence rate in TCGA, GES54460, GES70768, and MKSCC database.

Dataset	Clinical parameter					

	Recurrence			No recurrence		
		
	1 year	3 years	5 years	1 year	3 years	5 years
TCGA (*n* = 489)	29 (5.93%)	67 (17.30%)	81 (16.56%)	460 (94.07%)	422 (82.70%)	408 (83.44%)
GES54460 (*n* = 106)	24 (22.64%)	45 (42.45%)	51 (48.11%)	82 (77.36)	61 (57.55%)	55 (51.89%)
GES70768 (*n* = 111)	8 (7.20%)	16 (14.41%)	19 (17.11%)	103 (92.79%)	95 (85.59%)	92 (82.89%)
MKSCC (*n* = 129)	15 (11.63%)	28 (21.71%)	31 (24.03%)	114 (88.37%)	101 (78.29%)	98 (75.97%)

**FIGURE 3:**
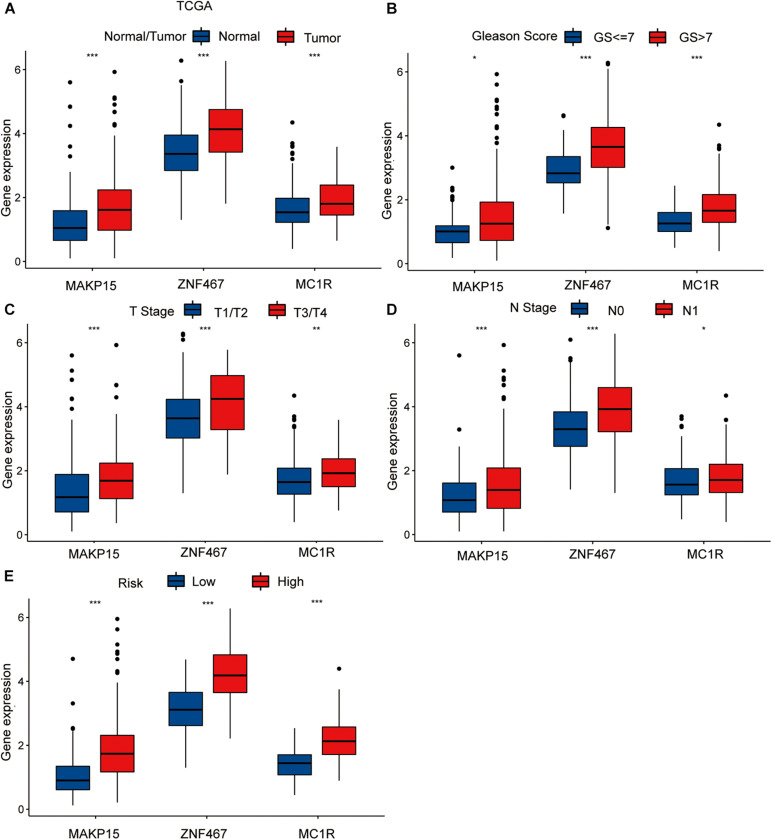
The differential analysis of three risk model genes between PRAD tissue and normal prostate tissue in TCGA **(A)**. The differential analysis of three risk model genes between different Primary Tumor T stage samples **(B)**, different Lymph Nodes N stage samples **(C)**, different Gleason score PRAD samples **(D),** and different risk samples **(E)** in TCGA cohort. The *P* values are labeled above each boxplot with asterisks (ns: represents no significance, *P < 0.05, **P < 0.01, ***P < 0.001).

To explore the single predictive value of the three-gene signature, we visualized the differential expression of each gene in samples with different clinical features. These included normal tissue and tumor samples, samples with T- and N-category characteristics, and samples differentiated according to their Gleason score and risk (TCGA dataset). Among these groups, those involving tumor samples, specifically category T3/T4 tumors, with N1 nodal status, high-risk, and Gleason score > 7 exhibited the highest gene expression levels (Figures 3A–E).

### Establishment and Appraisal of a Nomogram for DFS Prediction in PCa

We screened clinical variables with independent prognostic value using univariate and multivariate Cox regression analyses (Supplementary Table 3) and applied the screening results based on the Gleason score, T-stage, and risk score to construct a nomogram (Supplementary Figure 3D). We then assessed the clinical significance of the nomogram using ROC analysis and C-index calculations. The respective AUC values of the nomogram for 1-, 3-, and 5-year survival were 0.780, 0.783, and 0.777, respectively. Compared to the individual clinical variables, the nomogram exhibited superior predictive ability, suggesting that the clinical nomogram offered better predictive value in the prognosis of patients with PCa than the individual clinical variables or risk score models (Supplementary Figures 5A–H).

### Exploration of the Functions of eRNAs and Target Genes

By comparing the gene expression levels in samples from the high- and low-risk groups of the training cohort, 609 DEGs (| FC| ≥ 1, adjusted *P* < 0.05) were further analyzed. GO enrichment analysis was used to explore the functions of DEGs in high- and low-risk groups, and 130 GO terms were obtained (*P* < 0.05). The results showed that the GO functions with significant DEG enrichment were “humoral immune response,” “complement activation, classical pathway,” “humoral immune response mediated by circulating immunoglobulin,” “immunoglobulin mediated immune response,” “immunoglobulin complex,” and “B cell mediated immunity” (*P* < 0.01) (Supplementary Figure 6C and Supplementary Table 4).

GSEA was used to explore the potential signaling pathways that influence the risk score model in the high- and low-risk groups (*P* < 0.05). In the high-risk group, the highest-ranking signaling pathways were base excision repair, DNA replication, Drug metabolism—other enzymes, homologous recombination, and spliceosome. In the low-risk group, the highest-ranking signaling pathways were adherens junction, propanoate metabolism, sphingolipid metabolism, tryptophan metabolism, and valine, leucine, and isoleucine degradation (Supplementary Table 5 and Supplementary Figures 6A,B).

### Comparison of TMEs Between High- and Low-Risk Groups

Based on the GO enrichment analysis results, the genes included in the risk model may affect the occurrence and development of tumors, through their impact on certain immune-related functions such as immunoglobulin complex. Therefore, we compared the tumor microenvironments (TMEs) in the high- and low-risk groups.

**FIGURE 4:**
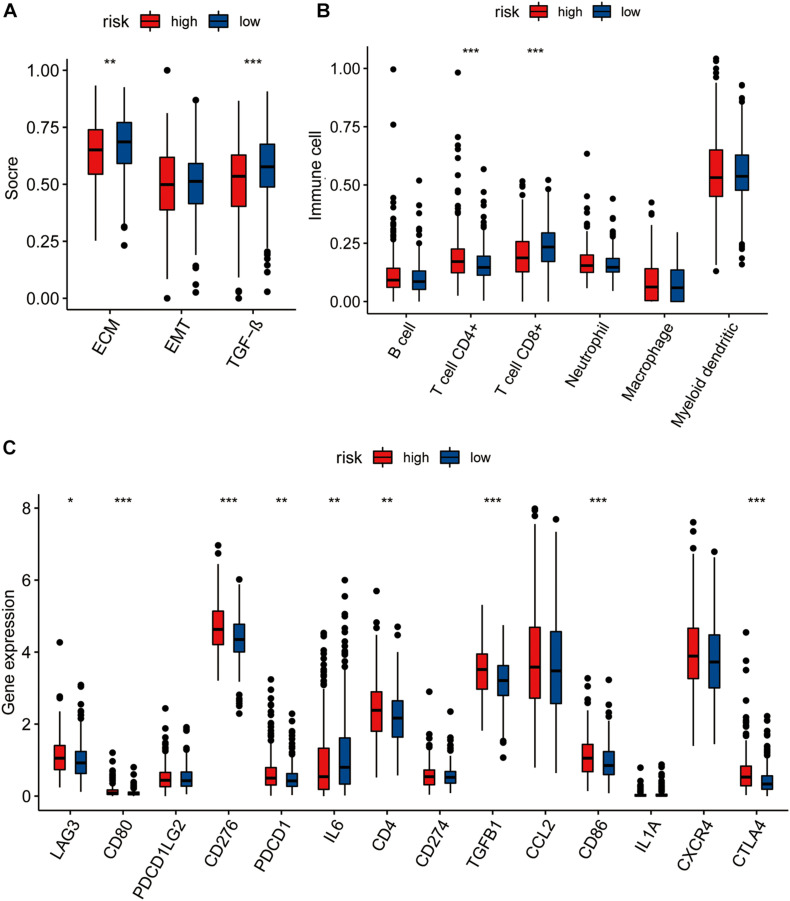
**(A)** The pairwise comparison of the ssGSEA score of EMT ECM, and TGF-b between high- and low-risk group. **(B)** The differential analysis of the abundance of immune cells between high-low risk groups. **(C)** The differential analysis of checkpoints between high-low risk groups. The *P* values are labeled above each boxplot with asterisks (ns: represents no significance,*P < 0.05, **P < 0.01, ***P < 0.001).

We calculated the single-sample GSEA (ssGSEA) scores of EMT, TGF-β, and ECM to compare the differences between the high- and low-risk groups, based on stromal cells and showed that the ECM and TGF-β scores of the low-risk group were higher than those of the high-risk group. No significant difference was seen in the EMT score between the high- and low-risk groups (Figure 4A).

We further explored immune cell infiltration to determine the immune status of the high- and low-risk groups and showed that CD4^+^ T cell and CD8^+^ T cell counts differed significantly between the two groups (Figure 4B). In addition to the TIMER algorithm, we used epic and xcell algorithms to compare the degree of immune infiltration in the two groups. The results of the EPIC algorithm showed that cancer associated fibroblast, T cell CD4+, T cell CD8+, macrophage, and uncharacterized cells were significantly different (Supplementary Figure 7A). The results of the xcell algorithm show that T cell CD4+ central memory, T cell CD4+ effector memory, T cell CD8+, T cell CD8+ effector memory, eosinophil, cancer associated fibroblast, neutrophil, T cell NK, plasmacytoid dendritic cell, immune score, and stroma scores were significantly different between the high and low risk groups (Supplementary Figure 7B).

We also compared the levels of expression of 14 immune checkpoints between the high- and low-risk groups and showed that the expression of nine of these immune checkpoints (LAG3, CD80, CD276, PDCD1, IL6, CD4, TGFB1, CD86, CTLA4) were significantly different between the high- and low-risk groups (Figure 4C). All except IL6 showed higher expression in the high-risk group than in low-risk group.

### Comparison of the DNA Mutation Burden, TMB, and MSI Between High- and Low-Risk Groups

Based on the GSEA results, base excision repair was associated with the heterogeneity between the high- and low-risk groups. We then explored the differences in the DNA mutation burden, tumor mutational burden (TMB), and microsatellite instability (MSI), between the two groups.

**FIGURE 5:**
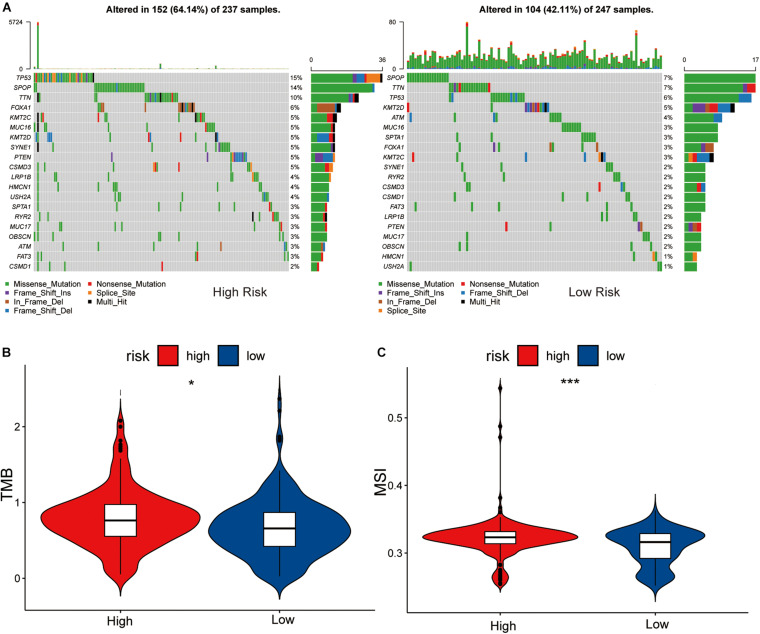
Association between high-low risk groups and DNA mutation. **(A)** The waterfall plot of the top 20 genes of DNA mutation in high-low risk groups. The pairwise comparison of TMB **(B)** and MSI **(C)** between high-low risk groups. The *P* values are labeled above each boxplot with asterisks (ns: represents no significance, *P < 0.05, ***P < 0.001).

By analyzing the 20 genes with the highest mutation frequency, we found a marked difference in mutation burden between the high- and low-risk groups. The genes with the highest mutational frequency in the high- and low-risk groups, were TP53 and SPOP, respectively (Figure 5A).

Comparing the differences in TMB between the high- and low-risk groups showed that the TMB of the high-risk group was significantly different to that of the low-risk group (*P* < 0.05), and the sensitivity of the low-risk group to immunotherapy was lower than that of the high-risk group (Figure 5B).

Differential analysis of the MSI of each PRAD sample obtained from a previous study (Zhang et al., 2020) showed that the low-risk group had a lower level of MSI than the high-risk group (*P* < 0.05, Figure 5C). We considered this to be MSI high in the high-risk group and MSI low in the low-risk group, due to the tumor’s disrupted function during DNA damage repair which increased gene instability. This is consistent with the results of TMB in the high and low risk groups.

### Drug Sensitivity Analysis

**FIGURE 6:**
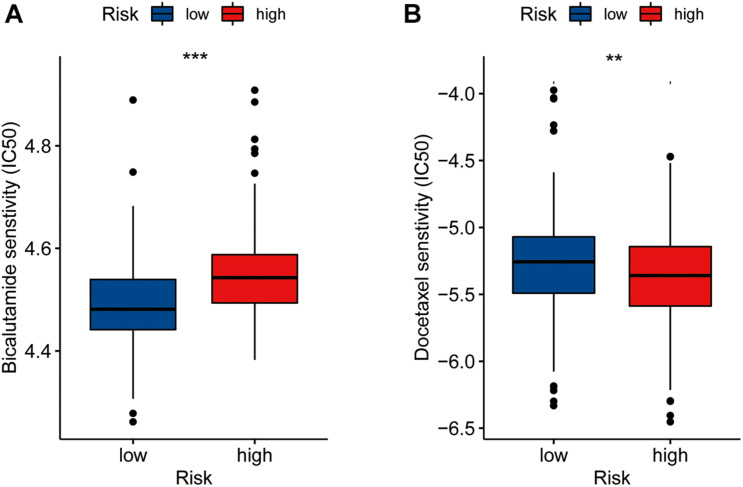
Drug sensibility analysis with risk model. Differences in the IC50 of five drugs [bicalutamide **(A)**, docetaxel **(B)**] in the high- and low-risk groups(**P < 0.01, ***P < 0.001).

We assessed differences in drug sensitivity between the high- and low-risk groups by analyzing the IC50 of two chemotherapeutic agents and showed that, among the drugs for which significantly different sensitivities were perceived between the high- and low-risk groups, patients in the high-risk group were more sensitive to docetaxel. Conversely, patients in the low-risk group were comparatively more sensitive to bicalutamide (Figures 6A,B). Through online CMap analysis, we identified 10 of the most significant small molecule candidate drugs that could improve prognosis in PCa, of which megestrol and tiletamine were the most likely to favorably alter gene expression (Supplementary Table 6).

## Discussion

Genomic and epigenomic changes in tumor cells are associated with certain tumor factors, including cellular proliferation and oncogenic transformation (Rhie et al., 2016). In recent years, several studies have illustrated the importance of lncRNAs (including eRNAs) in regulating gene transcription and protein synthesis (Ouyang et al., 2014; Engreitz et al., 2016). Compared with other types of lncRNA, eRNAs are ideal anticancer targets, as they affect the occurrence of many cancers by regulating the expression of multiple genes (van Velzen et al., 2020). However, few reports have described the influence of eRNAs on the development of PCa. Therefore, we identified eRNAs and eRNA-driven genes related to the prognosis of patients with PCa, to explore the possible mechanisms of these eRNAs’ influence on the occurrence and development of PCa. Our model showed positive values for the coefficients of the relevant three genes, indicating that these genes are promoters of PCa. Thus, we inferred a direct correlation between expression of the signature genes and risk of poor prognosis in PCa.

Through functional analysis, we found that the eRNAs and corresponding eRNA-driven genes may affect tumor development through certain immune processes. Previous studies have shown that immune cells are important in hindering tumor progression, and that the TME plays a key role in the occurrence and development of PCa and other cancers (Fortis et al., 2017; Togashi et al., 2019). For example, natural killer cells are regulated by the TME in the process of killing lung cancer tumor cells (Ren et al., 2015). Previous studies have also shown that ECM, TGF-β, and EMT are important factors affecting the TME and, resultantly, tumor progression (Park et al., 2019; Yang et al., 2020). In our study, EMT played no significant role in PCa progression. Fibrogenesis of ECM can promote tumor development (Barry et al., 2020). In the high-risk group of our study, this process might have been exaggerated, resulting in a poor prognosis. The TGF-β signaling pathway can inhibit tumor development by inducing the expression of multiple tumor-suppressor genes (Papoutsoglou and Moustakas, 2020). In this study, the function of the tumor-suppressor arm of the TGF-β signaling pathway might have been disabled in the high-risk group, thereby altering gene expression in a direction that is favorable to tumor development. Our study also revealed that the levels of CD8^+^ T cells were decreased in the high-risk group, whereas levels of CD4^+^ T cells were increased in the low-risk group. This suggests that patients in the high-risk group were in a state of immunodepletion and that the three genes of our model may promote tumor progression by accelerating immunodepletion. The high expression of immune checkpoint genes is an important mechanism of immune evasion in tumorigenesis (Zhang and Zheng, 2020). In our study, the levels of expression of most immune checkpoint genes were lower in the low-risk group than in the high-risk group, which may partially explain the poor prognosis of patients in the high-risk group. Thus, we can surmise that the three signature genes we have identified in this study may contribute to cancer development by increasing immune checkpoint expression.

The results of the functional analysis indicated that the eRNAs and corresponding eRNA-driven genes also influence the progression of PCa by affecting the base excision repairp rocess. We compared the tumor mutation spectrum, TMB, and MSI between the high- and low-risk groups and showed that the gene with the highest mutation frequency in the high-risk group was TP53, whereas in the low-risk group it was SPOP. TP53 mutations, which can significantly promote tumor growth, are consequentially associated with the development of PCa (Kaur et al., 2019), while SPOP is an important tumor-suppressor gene in PCa cells (Geng et al., 2017). Our results also showed that the high expression levels of eRNAs and corresponding eRNA-driven genes were associated with PCa prognosis, and could promote occurrence of TP53 mutations, which further aids the progression of PCa. It has been shown that MSI is associated with many genetic diseases and is a key indicator of genomic instability (Niu et al., 2014). TMB, defined as the total number of somatic gene coding errors, base substitutions, and gene insertion or deletion errors detected per million bases, is closely related to tumor heterogeneity (Merino et al., 2020; Salem et al., 2020). The GSEA results showed that the scores of TMB and MSI in the high-risk group were higher than those in the low-risk group, suggesting that the eRNAs and corresponding eRNA-driven genes could affect the process of base excision repair. We considered this to be MSI high in the high-risk group and MSI low in the low-risk group, due to the tumor’s disrupted function during DNA damage repair leading to increased gene instability, which is consistent with the results of TMB in the high and low risk groups. Jiang et al. (2021) found that patients with high TMB had worse survival outcomes than those with low TMB. We believe that this finding may account for the longer DFS in the low-risk group, compared to the high-risk group. In addition, TMB and MSI are closely associated with the efficacy of immunotherapy and can be used as biomarkers of immunotherapy response in PCa (Merino et al., 2020; van Velzen et al., 2020). Although PCa is an immunodesert tumor, immunity has a significant influence (Bilusic et al., 2017). Moreover, our GO enrichment results also showed that the DEGs in the high and low risk groups were related to immunity, which could explain the immune desertification of PCa. In our study, the high-risk group exhibited high immune cell infiltration and high immune checkpoint expression, suggesting that patients in this group could significant benefit from immune-targeted therapy. This finding was consistent with our results related to TMB and MSI. Therefore, our calculated risk scores were highly correlated with the prognosis of patients with PCa and the efficacy of immunotherapy. At present, drug therapy is used to a clinical setting for the treatment of PCa, mainly docetaxel and bicalutamide. The results of drug sensitivity analysis have shown that our model, based on the eRNA-driven genes, can better predict the drug sensitivity of patients with PCa, which could guide the selection of appropriate clinical drugs to a certain extent.

Based on the outcomes of our study, our method of constructing a risk score model is more reliable in producing a robust model than existing methods, and that our study was well-founded as the mechanisms by which eRNAs affect the occurrence and development of PCa, as well as their involvement in potential therapeutic options, were explored on many levels and through various methods. However, our study has some limitations. Firstly, the data were not from our own database but rather retrieved from public databases. Secondly, the mechanisms of action of the eRNAs and target genes in PCa, were not further investigated through *in vitro* and *in vivo* experiments.

## Conclusion

In summary, we used expression profiles from public databases to screen genes closely associated with the prognosis of patients with PCa and established a prognostic risk model, which was validated in multiple datasets. Differential analyses showed significant differences in TME, TMB, and MSI between the high- and low-risk groups. The genes included in the three-gene signature may affect tumor progression, by impacting the aforementioned factors. Therefore, the risk score derived from our model is a good predictor of the prognosis of patients with PCa and can be used to explore how eRNAs affect the occurrence and development of PCa. Drug sensitivity analysis showed that respondents in the high- and low-risk groups differed in their sensitivity to several chemotherapeutic agents used to treat PCa. Our model could predict drug sensitivity, and several candidate drugs that may inhibit the progression of PCa were identified. These findings can help to design personalized medication regimes for patients and exploring more effective drug options for PCa treatment.

## Data Availability Statement

The datasets presented in this study can be found in online repositories. The names of the repository/repositories and accession number(s) can be found in the article/Supplementary Material.

## Author Contributions

SF, ZW, and LZ designed the study, analyzed the data, revised the images, performed the literature search, and collected data for the manuscript. CZ, JW, and DY revised the manuscript. All authors contributed to the article and approved the submitted version.

## Conflict of Interest

The authors declare that the research was conducted in the absence of any commercial or financial relationships that could be construed as a potential conflict of interest.

## Footnotes

1 https://portal.gdc.cancer.gov/

2 http://www.ncbi.nlm.nih.gov/geo/

3 http://www.cbioportal.org/

4 http://www.cistrome.org/

5 https://www.gsea-msigdb.org/

6 http://software.broadinstitute.org/gsea/index.jsp

7 https://portals.broadinstitute.org/cmap/

8 https://www.uniprot.org/

## Supplementary Material

Supplementary Figure 1 Expression relationships of six eRNAs and corresponding eRNAs-driven Genes.

Supplementary Figure 2 KM survival curves for six eRNAs and nine target genes.

Supplementary Figure 3 Correlation analysis of three model genes’ copy number variation regions with target genes [MAPK15 **(A)**, ZNF467 **(B)**, and MC1R **(C)**].

Supplementary Figure 4 Time-dependent ROC curve analysis of the model genes [MAPK15 **(A)**, MC1R **(B)**, and ZNF467 **(C)**] in the TCGA cohort. Correlation analysis of three genes in the model **(D)**.

Supplementary Figure 5 The correlation of riskscore and Gleason score (≤7, >7) **(A)**, Lymph nodes (N0, N1) **(B)**, and T-stage (T1&T2, T3&T4) **(C)** expression. Nomogram to predict the 1-, 3- and 5-year DFS of PCa patients **(D)**. Concordance index of the indicated prognostic model in the training datasets **(E)**. Time-dependent ROC analysis was used to evaluate the accuracy of the DFS nomograms **(F–H)**. The red, blue, green, and orange solid lines represent the T-stage, gleason-score, risk-score, and total-points, respectively.

Supplementary Figure 6 The rows and columns indicate the genes and tumor samples, respectively. Enrichment plots of the top five KEGG pathways in the high-risk score **(A)** and low-risk score **(B)** groups in PRAD. GO analysis of differential expression genes in high-low risk group **(C)**.

Supplementary Figure 7 Analysis of the difference of immune infiltration between the two algorithms of epic **(A)** and xcell **(B)** in the high- and low-risk groups.

Supplementary Table 1 In 1,000 iterations, eRNAs and target genes appeared more than or equal to 700 times.

Supplementary Table 2 Functional markers of nine eRNAs-driven genes.

Supplementary Table 3 Clinical factors were analyzed by univariate and multivariate Cox.

Supplementary Table 4 130 GO terms of differential expression genes.

Supplementary Table 5 GSEA results of the KEGG pathways in the high-risk score and low-risk score groups in PRAD.

Supplementary Table 6 10 Important Small Molecule Drugs that may reverse tumor status of PCa.

## Abbreviations

PCa: Prostate cancer
eRNAs: Enhancer RNAs
lncRNAs: Long non-coding RNAs
SCCHN: head and neck squamous cell carcinoma
GO: Gene ontology
GSEA: Gene Set Enrichment Analysis
DEGs: differentially expressed genes
TCGA: The Cancer Genome Atlas
NIH: National Institutes of Health
GDC: Genomic Data Commons
RNA-seq: RNA sequencing
GEO: Gene Expression Omnibus
TIMER2.0: Tumor Immune Estimation Resource 2.0
PRAD: prostate adenocarcinoma
GDSC: Genomics of Drug Sensitivity in Cancer
EMT: Epithelial-mesenchymal transformation
TGF- β: transforming growth factor β
ECM: extracellular matrix
MSigDB: Molecular Signatures Database
DFS: disease-free survival
KM: Kaplan–Meier
LASSO: least absolute shrinkage and selection operator
ROC: receiver operator characteristic
AUC: area under the curve
FDR: false discovery rate
FC: fold change
C-index: concordance index
TME: tumor microenvironment
TMB: tumor mutational burden
MSI: microsatellite instability
IC50: half-inhibitory concentration.
