# Supplementary figures and images for: A Robust Prognostic Gene Signature Based on eRNAs-Driven Genes in Prostate Cancer
Source: Front Genet. 2021 Jun 29;12:676845. doi: 10.3389/fgene.2021.676845 (PMC8276043; doi:10.3389/fgene.2021.676845)

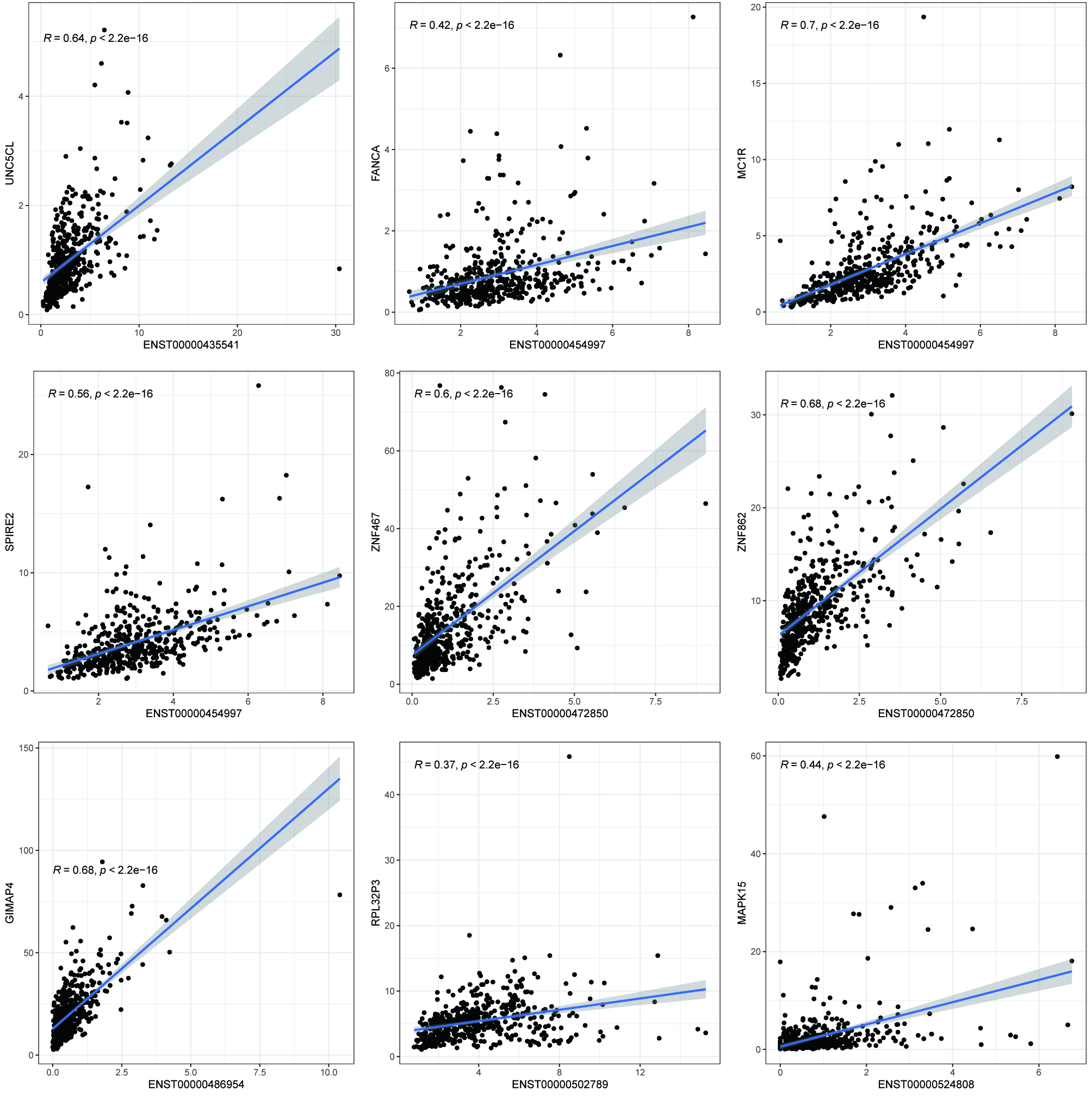

Supplement: Supplementary Figure 1 — Expression relationships of six eRNAs and corresponding eRNAs-driven Genes. [file Image_1.TIF]

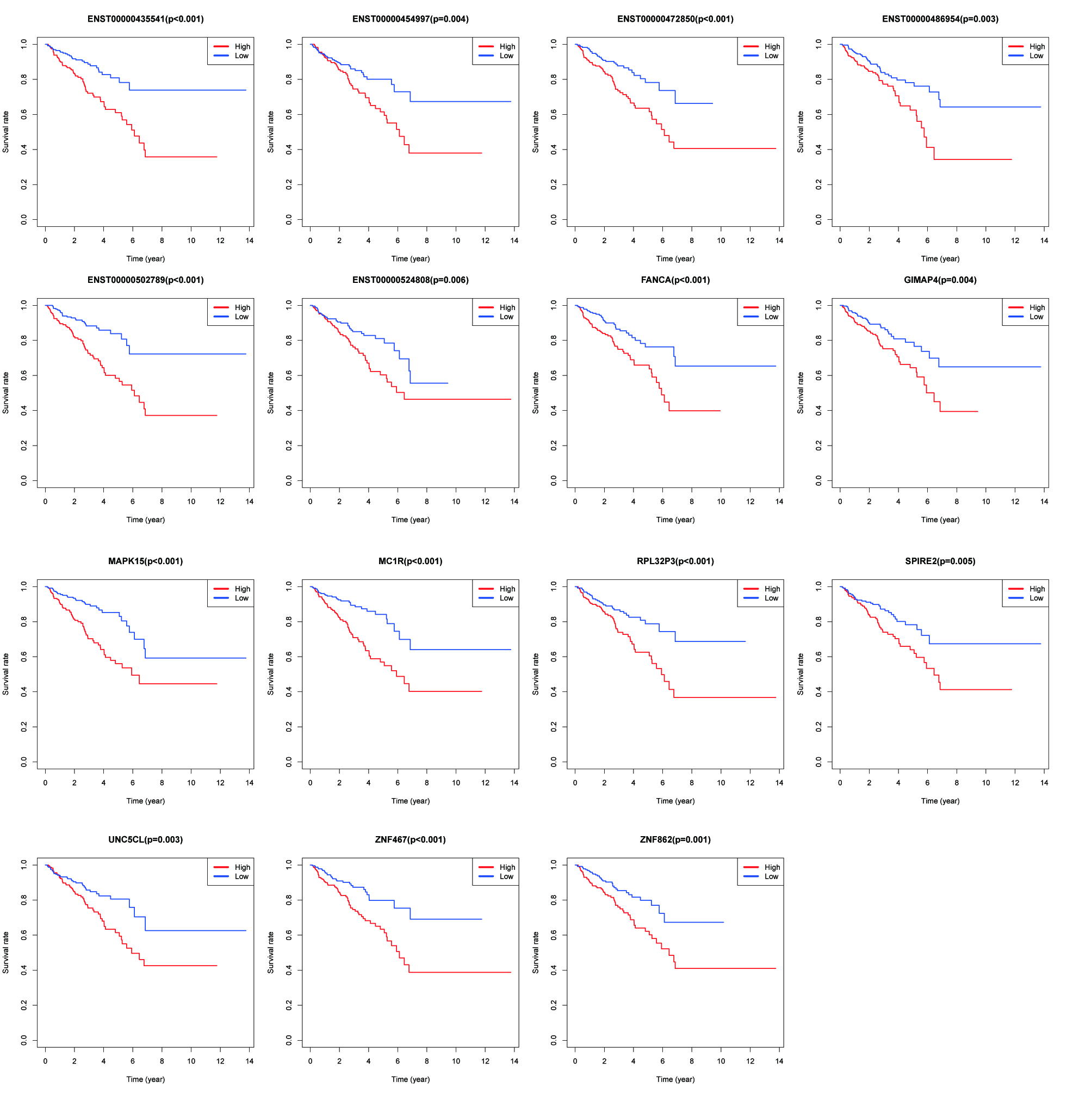

Supplement: Supplementary Figure 2 — KM survival curves for six eRNAs and nine target genes. [file Image_2.TIF]

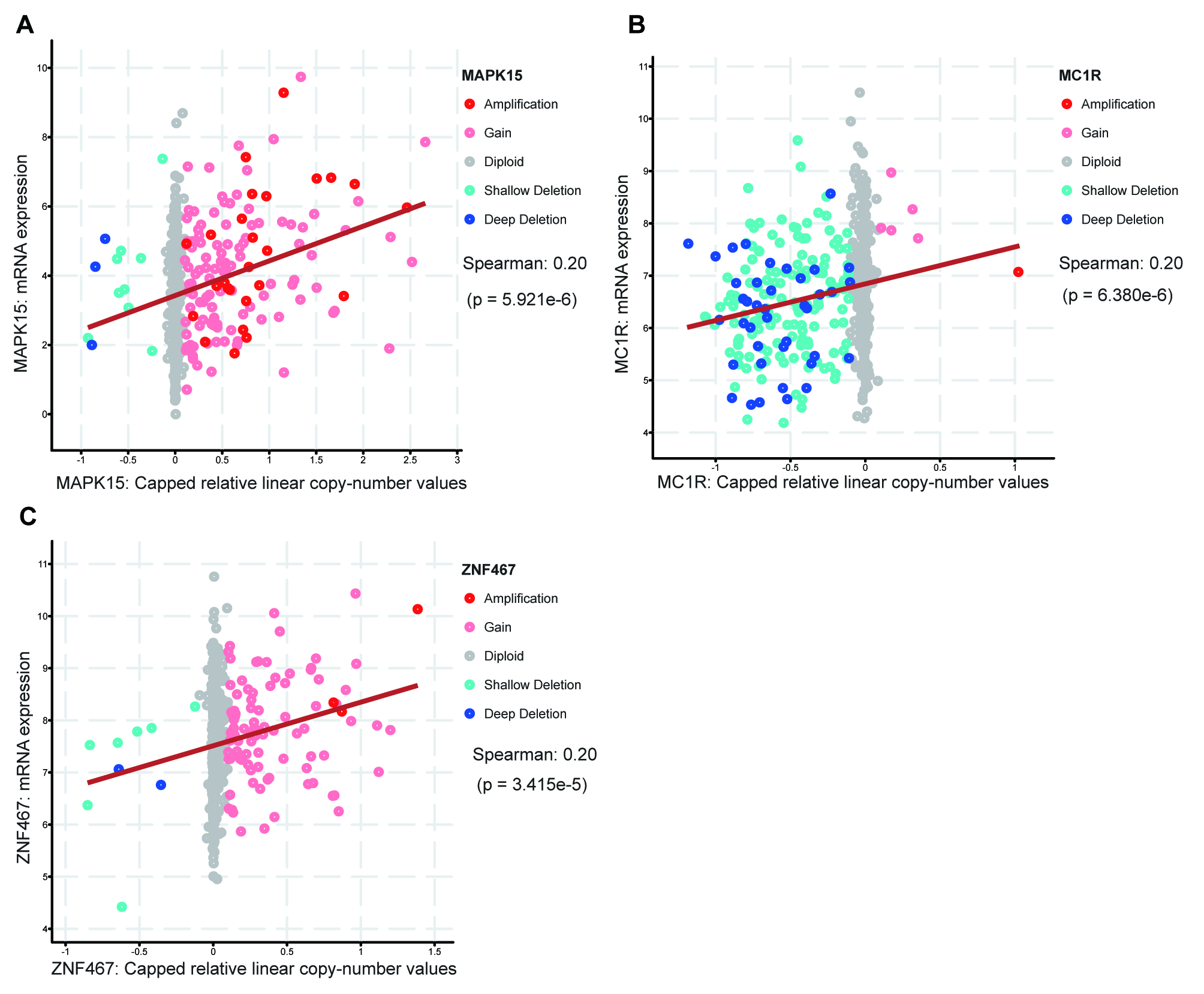

Supplement: Supplementary Figure 3 — Correlation analysis of three model genes’ copy number variation regions with target genes [MAPK15 (A), ZNF467 (B), and MC1R (C)]. [file Image_3.TIF]

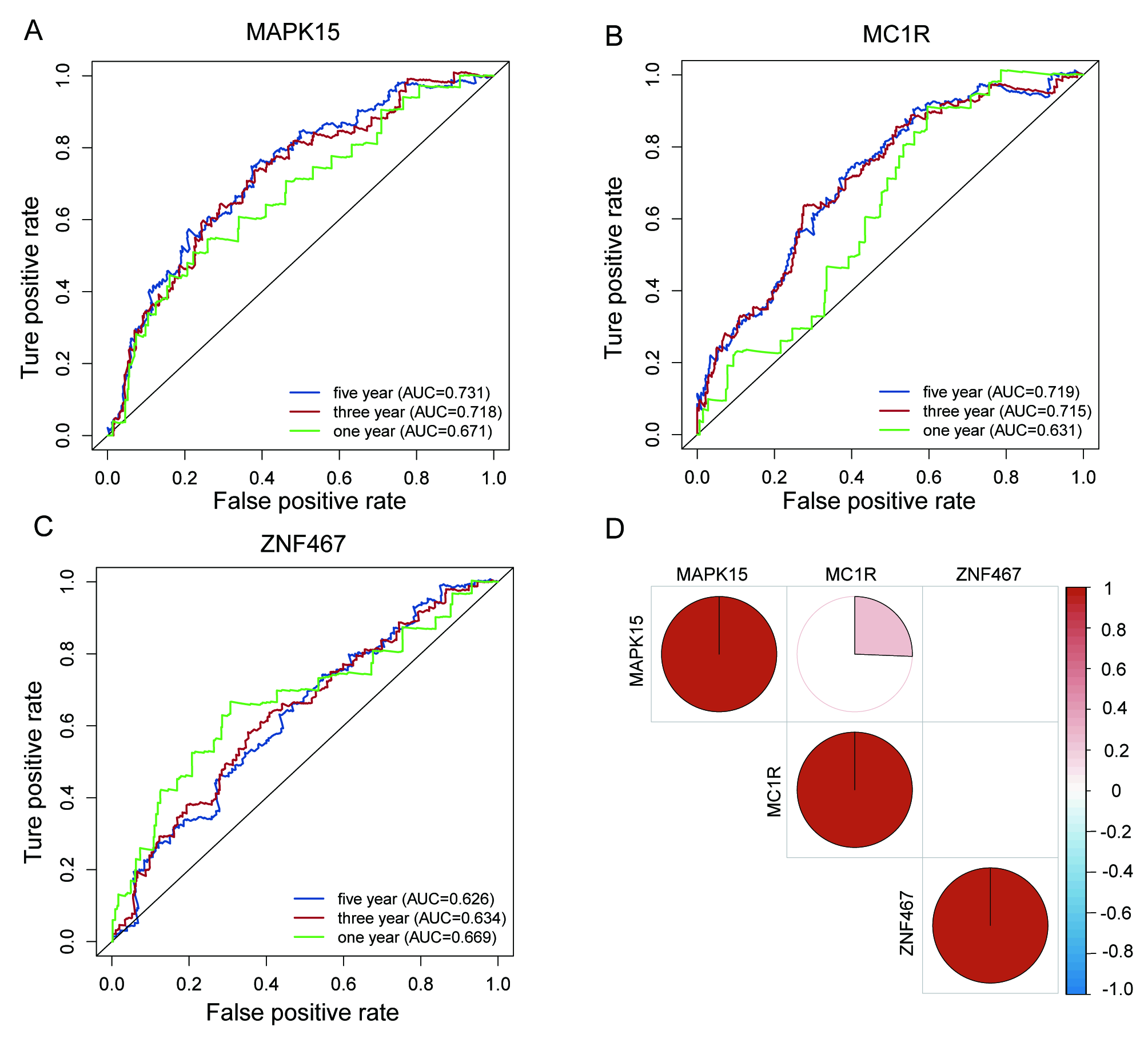

Supplement: Supplementary Figure 4 — Time-dependent ROC curve analysis of the model genes [MAPK15 (A), MC1R (B), and ZNF467 (C)] in the TCGA cohort. Correlation analysis of three genes in the model (D). [file Image_4.TIF]

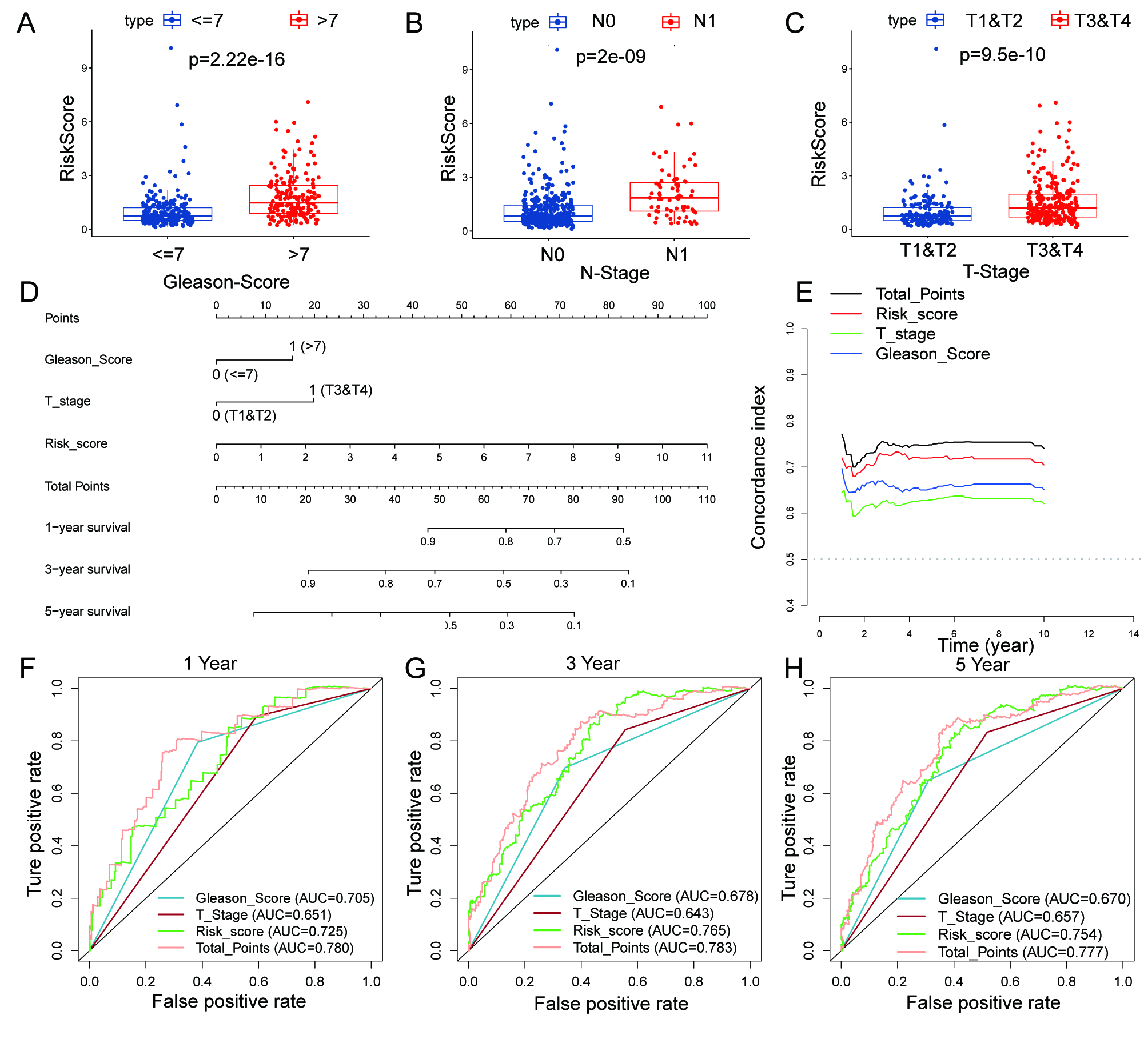

Supplement: Supplementary Figure 5 — The correlation of riskscore and Gleason score (≤7, >7) (A), Lymph nodes (N0, N1) (B), and T-stage (T1&T2, T3&T4) (C) expression. Nomogram to predict the 1-, 3- and 5-year DFS of PCa patients (D). Concordance index of the indicated prognostic model in the training datasets (E). Time-dependent ROC analysis was used to evaluate the accuracy of the DFS nomograms (F–H). The red, blue, green, and orange solid lines represent the T-stage, gleason-score, risk-score, and total-points, respectively. [file Image_5.TIF]

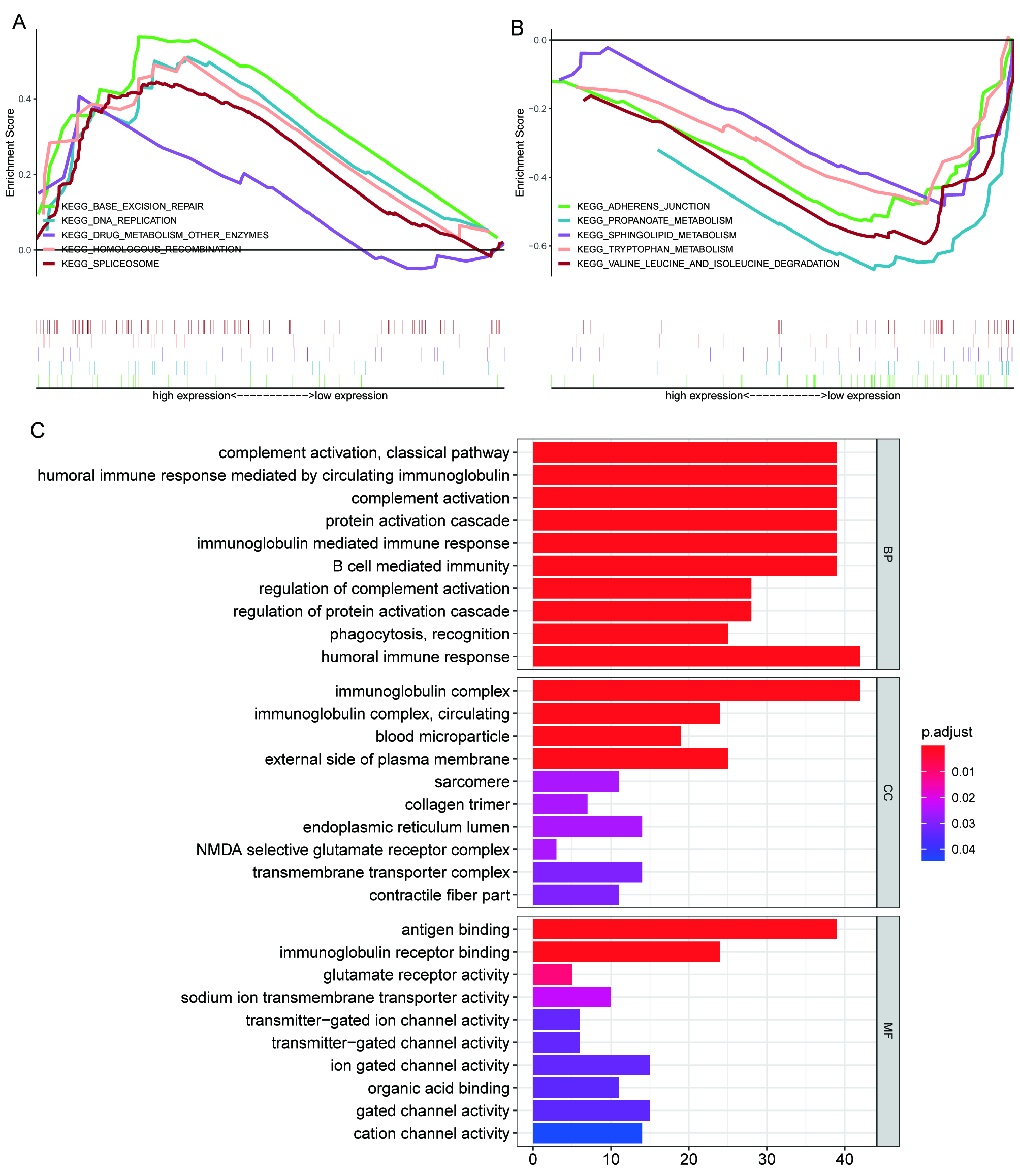

Supplement: Supplementary Figure 6 — The rows and columns indicate the genes and tumor samples, respectively. Enrichment plots of the top five KEGG pathways in the high-risk score (A) and low-risk score (B) groups in PRAD. GO analysis of differential expression genes in high-low risk group (C). [file Image_6.TIF]

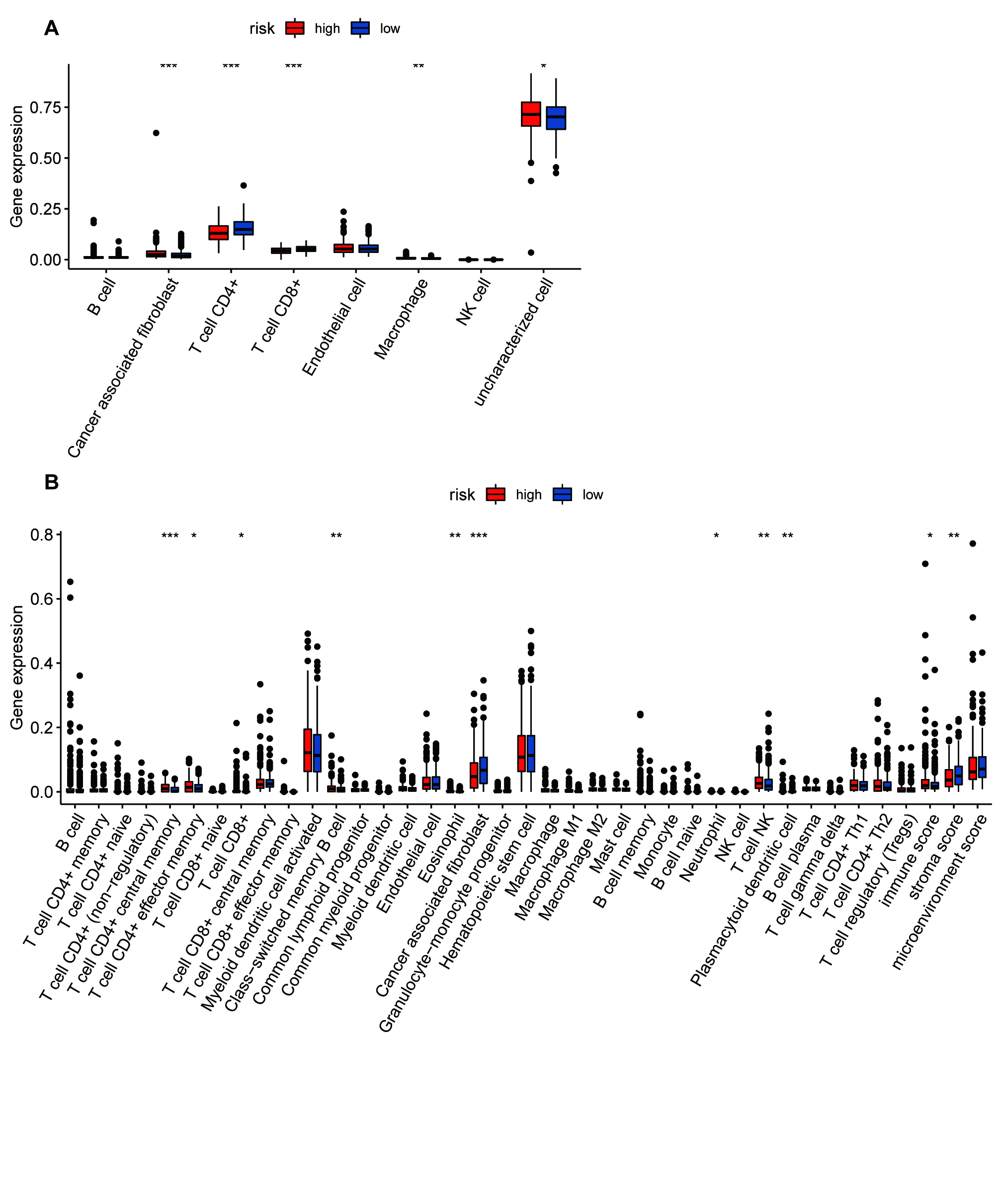

Supplement: Supplementary Figure 7 — Analysis of the difference of immune infiltration between the two algorithms of epic (A) and xcell (B) in the high- and low-risk groups. [file Image_7.TIF]
